# Lignin accumulation in cell wall plays a role in clubroot resistance

**DOI:** 10.3389/fpls.2024.1401265

**Published:** 2024-07-23

**Authors:** Jiangying Tu, Li Qin, Chithra Karunakaran, Yangdou Wei, Gary Peng

**Affiliations:** ^1^ Saskatoon Research and Development Centre, Agriculture and Agri-Food Canada, Saskatoon, SK, Canada; ^2^ Department of Biology, University of Saskatchewan, Saskatoon, SK, Canada; ^3^ Canadian Light Source Inc., University of Saskatchewan, Saskatoon, SK, Canada

**Keywords:** *Brassica napus*, *Plasmodiophora brassicae*, clubroot resistance, exodermis, lignin, cell wall alteration, fourier transform mid-infrared microspectroscopy

## Abstract

Clubroot, caused by *Plasmodiophora brassicae*, is a significant disease affecting brassica crops worldwide and poses a threat to canola (*Brassica napus*) production in western Canada. Management of this disease heavily relies on the use of resistant cultivars, but resistance erosion is a serious concern due to the highly diverse pathogen populations. Understanding resistance mechanisms may aid in better deployment/rotation of clubroot resistance (CR) genes and improve resistance resilience. In this study, we conducted a comparative analysis using resistant canola varieties carrying either a single (*Rcr1*) or double CR genes (*Rcr1*+*Crr1^rutb^*) to decipher the resistance modes associated with these genes. Cell wall (CW) biopolymeric compounds in different root layers were mapped and quantified using Fourier-transform mid-infrared microspectroscopy for changes in CW elements associated with clubroot resistance. Transmission electron and confocal microscopy were used to assess root infection details and relative transcript abundance was analyzed to determine the activation of the lignin-related pathway in relation to resistance. Neither resistant variety affected the primary infection of root hairs/epidermal cells compared to the susceptible “Westar”, but both exhibited strong inhibition of cortical infection, effectively ‘trapping’ the pathogen in the exodermis. The most prominent change observed was increased lignin accumulation associated with resistance. In Westar, the pathogen was able to degrade CW lignin, facilitating access to the root cortex by secondary plasmodia of *P*. *brassicae*. In contrast, resistant varieties showed clear lignin accumulation around the penetration site on the exodermis, accompanied by elevated expression of genes involved in the phenylpropanoid pathway. These results suggest that induced lignin accumulation plays a role in clubroot resistance mediated by the CR genes *Rcr1* and *Crr1^rutb^* in canola, providing cellular and structural evidence that supports the data from earlier transcriptomic studies.

## Introduction

*Plasmodiophora brassicae* Woronin, a soil-borne protist, is the causal agent of clubroot disease in the *Brassicaceae* family, including canola/rapeseed and vegetable crucifers. Early infection can lead to serious crop yield loss (Strehlow et al., 2015; Javed et al., 2023). The pathogen has three distinct stages in its life cycle: i) Survival in the soil as resting spores, ii) primary infection occurring in root hairs and epidermal cells (Liu et al., 2022), and iii) secondary infection in the cortex leading to the hypertrophy of root cells and gall formation, during which the cell wall of infected tissue may become ‘loosened’. The gall formation also perturbs vascular development with a significant reduction in xylem, affecting the uptake of water and nutrients by the root, and increases in phloem bundles, facilitating sugar transport to the stimulated sink location (Malinowski et al., 2012; Walerowski et al., 2018). Ultrastructural observations have shown that the movement of secondary plasmodia from infected cells to neighboring cells is possibly via ‘openings’ in the cell wall (CW) and local dissolution of infected CWs with that of neighboring uninfected cell (Mithen and Magrath, 1992; Deora et al., 2013). Unlike other pathogens with many CW-related polysaccharide degrading enzymes, the *P. brassicae* genome encodes few carbohydrate-active or CW-degrading enzymes (Schwelm et al., 2015; Rolfe et al., 2016). It is possible that such a CW alteration to infected cells, which could function as a feeding site providing nutrients to *P. brassicae*, is mediated by induced regulation of host genes.

The plant CW consists of polysaccharides (cellulose, hemicellulose and pectins), proteoglycan (extensins and arabinogalactan proteins) and, in some cells, polyphenolics (lignin) and polyesters (cutin and suberin). CW modifications, induced frequently by infection, can alter the outcome of infection process, resulting in disease resistance (Hématy et al., 2009; Wieczorek, 2015; Houston et al., 2016; Bacete et al., 2018; Bozbuga et al., 2018; Molina et al., 2021). However, the role of CW polymers in plant defense against clubroot has not been unequivocally clear; previous studies based on transcriptome analysis reported alterations of CW-related gene expression associated with clubroot infection (Agarwal et al., 2011; Chu et al., 2014; Irani et al., 2018; Ciaghi et al., 2019; Badstöber et al., 2020; Wen et al., 2024). For example, the expression of genes encoding CW modification and hydrolytic enzymes changed substantially during the infection of susceptible *B. rapa*, *B. oleracea*, and Arabidopsis by *P. brassicae* (Agarwal et al., 2011; Irani et al., 2018; Badstöber et al., 2020). Transcription of genes involved in cellulose, hemicellulose, pectin and lignin biosynthesis were up-regulated in symptomless roots of *B. oleracea*, while CW modification and degradation genes were down-regulated (Ciaghi et al., 2019). With the progression of clubroot development in susceptible *B. napus*, CW lignification was reduced in infected tissues (Deora et al., 2013); genes involved in the lignification process were down-regulated (Cao et al., 2008). On the other hand, thickening and lignification of CWs were suggested to limit the spread of pathogen plasmodia in tolerant *B. oleracea* (Donald et al., 2008) and *B. rapa* (Song et al., 2016; Zhang et al., 2016; Lahlali et al., 2017; Yuan et al., 2021). Additionally, genes involved in the phenylpropanoid pathway contributing to the accumulation of lignin or phenolic metabolites were up-regulated in resistant *B*. *napus* and *B*. r*apa*, respectively (Irani et al., 2019; Yuan et al., 2021). However, it remains largely unknown how the re-localization and deposition of lignin occur at the cellular and tissue levels in response to *P*. *brassicae* infection.

CW remodeling during root infestation by parasitic nematodes was considered essential for the nematodes to complete their life cycle (reviewed by Bohlmann and Sobczak, 2014). However, it remains a question how CWs and their composition influence the spread and development of *P. brassicae* plasmodia during cortical infection. In this study, we aimed to investigate changes in CW components during the infection process of *P*. *brassicae* pathotype 3H to determine the role of phenolics and lignin in clubroot resistance, as indicated by prior transcriptomic studies (Chu et al., 2014; Wen et al., 2024). The pathotype 3H is the predominant *P. brassicae* strain in western Canada (Strelkov et al., 2018).

## Materials and methods

### Plant materials and growth condition

Two canola varieties, CPS13 (PS-FCA 15–3978) carrying the CR gene *Rcr1* on chromosome A03 and CPS14 (PS-ARK 14–3562) carrying the CR genes *Rcr1* and *Crr1^rutb^* (on A08) (Wen et al., 2024) were provided by the Nutrient Ag Solutions (Saskatoon, SK). Both varieties are hybrids. A DH population of “Westar” that carries no CR gene was used as a susceptible control. The seeds were germinated on moistened filter paper in the dark for two days before being transplanted in Sunshine #3 soilless potting mix (Sun Gro Horticulture Canada Ltd., Vancouver, BC). After transplantation, plants were maintained in a growth chamber (~ 23°/10°C, day/night) with a 14h photoperiod (230 μmol/m^2^/s).

### Inoculation and sampling

Seven days after transplantation, canola plants were inoculated with *P. brassicae* pathotype 3H (Williams’ differential system). Inoculum was prepared from frozen clubroot galls stored in -20°C freezers. The spore concentration was estimated using a hemocytometer and adjusted to 1.0×10^7^ spores/mL. For inoculation, 2.5 mL of spore suspension was applied to the base of seedlings by pipetting, and mock plants were treated similarly with water. Canola root samples were collected shortly after the inoculation (0-time point) and at 2, 5, 8, 11 and 15 days post inoculation (dpi), and washed with running water to remove soil particles from the surface. Root samples from 4 to 8 plants were pooled to form a biological replicate for DNA/RNA extraction. There were three biological replicates for each treatment at each time point. Additional plants were kept until five weeks after inoculation (35 dpi) and assessed for disease severity (Chu et al., 2014).

### Visualizing and quantifying *P. brassicae*

Root samples were collected at 3, 5, 7, 10 and 12 dpi, stained and visualized for infection using confocal microscopy (Tu et al., 2019). For *P. brassicae* biomass measurement, DNA was isolated from canola root samples collected at 0, 2, 5, 8, 11 and 15 dpi and quantified with qRT-PCR (quantitative reverse transcription PCR) using primers targeting *S17 Ribosome Protein* gene in the *ITS* of *P. brassicae* and the *ACTIN2* gene of *B*. *napus* (Jiang et al., 2020). The genomic DNA of *P. brassicae* and canola (*B*. *napus*) was quantified with qRT-PCR on the StepOne^®^ Plus cycler (Thermo Fisher Scientific, Waltham, MA) using SYBR Green (Qiagen, Mississauga, ON) and species-specific primers (**Supplementary Table 1**; Siemens et al., 2009; Rennie et al., 2011). Relative DNA content was calculated using the 2^-ΔΔCт^ method (Livak and Schmittgen, 2001).

### Transmission electron microscopy

Infected roots were sampled at 12 dpi when susceptible Westar started showing root swellings. Upper roots close to hypocotyl (approximately 1 cm long) were harvested and fixed overnight in a freshly prepared 2% glutaraldehyde (SPI Supplies, West Chester, PA) solution in 0.1 M phosphate buffer (PSB) at pH 7.4 overnight at 4°C, washed three times in fresh PSB and post-fixed in 1% osmium tetroxide (SPI Supplies), then in freshly prepared in 0.1 M PSB for 4 h followed by three washes in distilled water. Tissues were dehydrated through an ascending ethanol series (25%, 50%, 75%, 95%, 100%) and embedded in Epon 812 resin (SPI Supplies). Ultra-thin sections were made using a diamond knife (DiATOME US, Diatome, PA) on a Reichert-Jung microtome (Reichert Microscopic Service; Depew, NY) and collected onto 100 naked mesh copper grids. Sections were post-stained with 2% uranyl acetate for 30 min in the dark, followed by Reynolds lead citrate (Ted Pella, Inc. Redding, CA) for 10 min, and observed with a Hitachi HT7700 transmission electron microscopy. For anatomical analysis of inoculated root samples, sections were stained with aqueous toluidine blue (0.01% in 0.1% sodium tetraborate).

### FTIR imaging

Cryo-sections were prepared from inoculated and non-inoculated roots sampled at 12 dpi by flash-freezing them in liquid nitrogen, then embedding them in Tissue-Tek cryomolds with FSC 22 Frozen Section Media (Leica, Mississauga, ON) at -20°C. Embedded tissue blocks were trimmed, cross-sectioned (10-μm-thick) with a Leica CM1860 cryotome and mounted on CaF_2_ circle windows (Crystran Ltd, Poole, Dorset, UK). The FTIR microspectroscopic data were collected at the mid-infrared beamline (01B1–1) of the Canadian Light Source Inc. (Saskatoon, SK) using an Agilent Cary 670 FTIR spectrometer coupled with a Cary 620 microscope system. The system was equipped with a 25× objective, matching condenser (NA = 0.82), and a 128 × 128 pixel liquid nitrogen cooled focal plane array (FPA) detector. This configuration would allow a field of view of approximately 422.4 μm × 422.4 μm (3.3 μm pixel resolution/pixel area on the sample plane) and the simultaneous acquisition of 16,384 spectra. All spectra were collected between 3850 and 900 cm^-1^ with the co-addition of 64 scans at 4 cm^-1^ resolution. The background spectra were recorded from a sample-free area on the windows. The background calibration in an empty region of the CaF_2_ slide was repeated for each sample cross-section every measurement. Chemical mapping was performed using Agilent Resolutions Pro 5.4 software.

### FTIR data processing

The data was analyzed using the software Quasar (https://quasar.codes). To minimize baseline shifts and improve the resolution of complex bands, the spectra were corrected by performing a baseline correction (“Rubber band”) and subtracting the background. For spectrum interpretation, we used the literature information to annotate bands associated with CW biopolymers and lipids of our data (**Table 1**). The quantitative estimation of important CW compounds (lignin, pectin, cellulose and hemicellulose) and lipids was determined from integrated areas between the baseline and specific spectral bands using Quasar. For each peak, a false-color map was generated based on the integrated peak area with the same color scale applied to all samples to enable direct visual comparison between samples. The color maps were contrasted using a ‘rainbow’ color scheme available in Quasar (Advanced Orange), with red indicating the most substantial absorbance and blue the lowest. The corrected zero-order data were transferred into the second derivative form using the Savitzky-Golay algorithm (nine smoothing points) in Quasar to determine subtle spectral changes. An average spectrum was generated for each treatment from the zero-order and second-derivation replicates. Pre-processed data, including the spectra of replicates, integrated area values and the mean for each treatment, were exported from Quasar as a comma-separated values (CSV) file for statistical analysis.

**Table 1:** Assignment of bands in fourier transform mid infrared spectra of cell walls.

wavenumber (cm^-1^)	Corresponding biopolymeric compounds in cell walls ^a^
1750–1720	Pectin/polygalactouronic acid, ester, carbonyl C=O stretching
1615–1595	C=O skeletal vibrations (aromatic ring stretch) plus C=O stretch; S > G
1520–1505	C=C skeletal vibrations (aromatic ring stretch), G > S
1445–1400	(COO-) mainly from de-esterized pectins
1390–1350	CH_2_ stretch of cellulose
1340–1300	Lignin, aliphatic OH bend, S-ring plus G-ring condensed
1261–1200	C=O/C-O stretch for hemicellulose
1170–1140	C-O-C stretch from pyranose rings in the cellulose group
1130–1092	cellulose
1090–1005	C-O-C stretch from pyranose rings in the cellulose group

a For the assignment of FTIR peaks to different compounds refer to Largo-Gosens et al., (2014); Kumar et al. (2016); Lahlali et al. (2017); Türker-Kaya (2017); Bock and Gierlinger (2019); Brar et al. (2019), and Beć et al. (2020). Based on the literature, three band areas, including 1090–1005 cm-1, 1130–1092 cm^-1^ and 1170–1140 cm^-1^, can be assigned to cellulose or cellulose groups due to the presence of C-O vibrations. In our study, the IR spectra in the band area 1090–1005 cm^-1^ with a peak around 1060 cm^-1^ was dominant, and therefore was chosen as the indicator for cellulose in CW. Similarly.

### Lignin staining

To visualize lignin deposition, lateral root samples were collected at 12 dpi and stained with basic fuchsin (Sigma-Aldrich, Oakville, ON), as per the ClearSee protocol described by Ursache et al. (2018). Following staining, root samples were imaged using a LSM880 confocal microscope, employing 561 nm excitation and detecting fluorescence emissions within the 600–650 nm range specifically for basic fuchsin.

### Expression of genes involved in lignin biosynthesis over the time course of infection

Total RNA was extracted from inoculated and corresponding control root samples at 0, 5, 8, 11 and 15 dpi using the method described by Missihoun et al. (2011). cDNA was generated from 1 μg of total RNA using the QuantiTect Reverse Transcription Kit (Qiagen) and PCR conducted using the Power SYBR green master mix (Qiagen) following manufacturer’s instructions. Cycling conditions were 95°C for an initial 10 min followed by 40 cycles of 15 s at 95°C, 30 s at 60°C and finally 30 s at 72°C. Melt-curve profiling and agarose gel electrophoresis were conducted to determine the specificity of the reaction and absence of primer dimers. All primers used in this study were described in **Supplementary Table 1**. The 2^-ΔΔCт^ method was used to analyze relative transcript abundance. Gene expression was normalized to the endogenous housekeeping gene *ACTIN2*, with data at 0 dpi being used as a reference (Livak and Schmittgen, 2001).

### Data analysis

All statistical analyses were conducted using R within the RStudio 1.4.1717 (R Core Team, 2021). Principle component analysis (PCA) was performed for the 1800–900 cm^−1^ region of IR spectra using the STATS package; the prcomp() function in STATS was used with the scale option set as false, and the plot() function was used to generate PCA plots. Representative spectra for 1800–900 cm^−1^ region of IR were generated using the hyperSpec package in R (Beleites and Sergo, 2018).

The data on CW components revealed by mid-infrared integrated area were analyzed using the following pipeline: Data were assessed for their suitability using parametric tests based on normal distribution of residuals (Shapiro-Wilk tests, Shapiro and Wilk, 1965) and homogeneity of variance. Two-way ANOVA analyses with type II sum of squares were carried out using the ‘emmeans’ test of R-studio. Multiple pairwise comparisons between effects and varieties related to compound class and infection status of the host plants were calculated using the contrast function in the emmeans package (Lenth, 2019). Mean values of biomass and gene expression for lignin biosynthesis pathway were subjected to two-way ANOVA for each variety and time points, followed by a Tukey HSD *post hoc* test with multiple comparison extensions.

## Results

### Development of *P. brassicae* during primary infection

The canola varieties CPS13 and CPS14 were generally resistant to *P. brassicae* pathotype 3H (**Supplementary Figure S1**), with CPS14 (two CR genes) exhibiting the lowest disease index. Five weeks after inoculation, all Westar plants had developed large galls, while small swellings were occasionally observed on some CPS13 plants, but not on any of CPS14.

**Figure 1:**
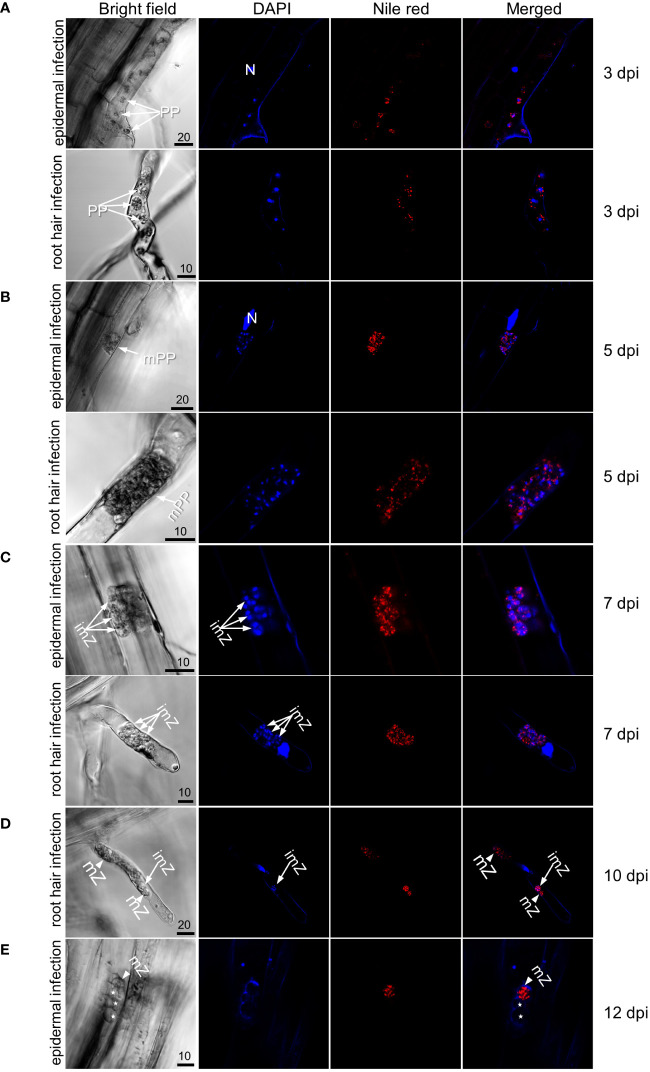
Observation of primary infection of Westar (susceptible) roots by *P*. *brassicae*. **(A)** Uni-nucleated primary plasmodia in which nuclei were stained by DAPI and lipids droplets stained by Nile red in an epidermal cell (top panel) and root hair (bottom panel) at 3 dpi. **(B)** Multi-nucleated primary plasmodia in an epidermal cell (top panel) and root hair (bottom panel) at 5 dpi. **(C)** Immature zoosporangia in a root hair at 7 dpi. **(D)** Mature zoosporangia in which nuclei were absent of DAPI staining and lipid droplets were stained by Nile red in a root hair at 10 dpi. **(E)** Empty zoosporangia in an epidermal cell at 12 dpi after releasing secondary zoospores. Labels N, plant nuclear; PP, uninucleate primary plasmodia; mPP, multinucleate primary plasmodia; imZ, immature zoosporangia; mZ, mature zoosporangia; asterisks indicate empty zoosporangia. Scale bar unit: μm.

Previous studies indicate that primary infection generally completes within two weeks after inoculation (Sharma et al., 2011; Liu et al., 2022; Javed et al., 2023). For verification in these canola varieties, inoculated roots were double-stained with DAPI and Nile red over a period of 3–12 dpi and examined with confocal microscopy (**Figure 1**). As shown in earlier studies, primary infection occurred in both susceptible and resistant varieties, and by 12 dpi, secondary zoospores were mostly released from infected root hairs or epidermal cells, evidenced by empty zoosporangia which were not stained by either DAPI or Nile red (**Figures 1D, E**).

### Development of *P. brassicae* during secondary infection

**Figure 2:**
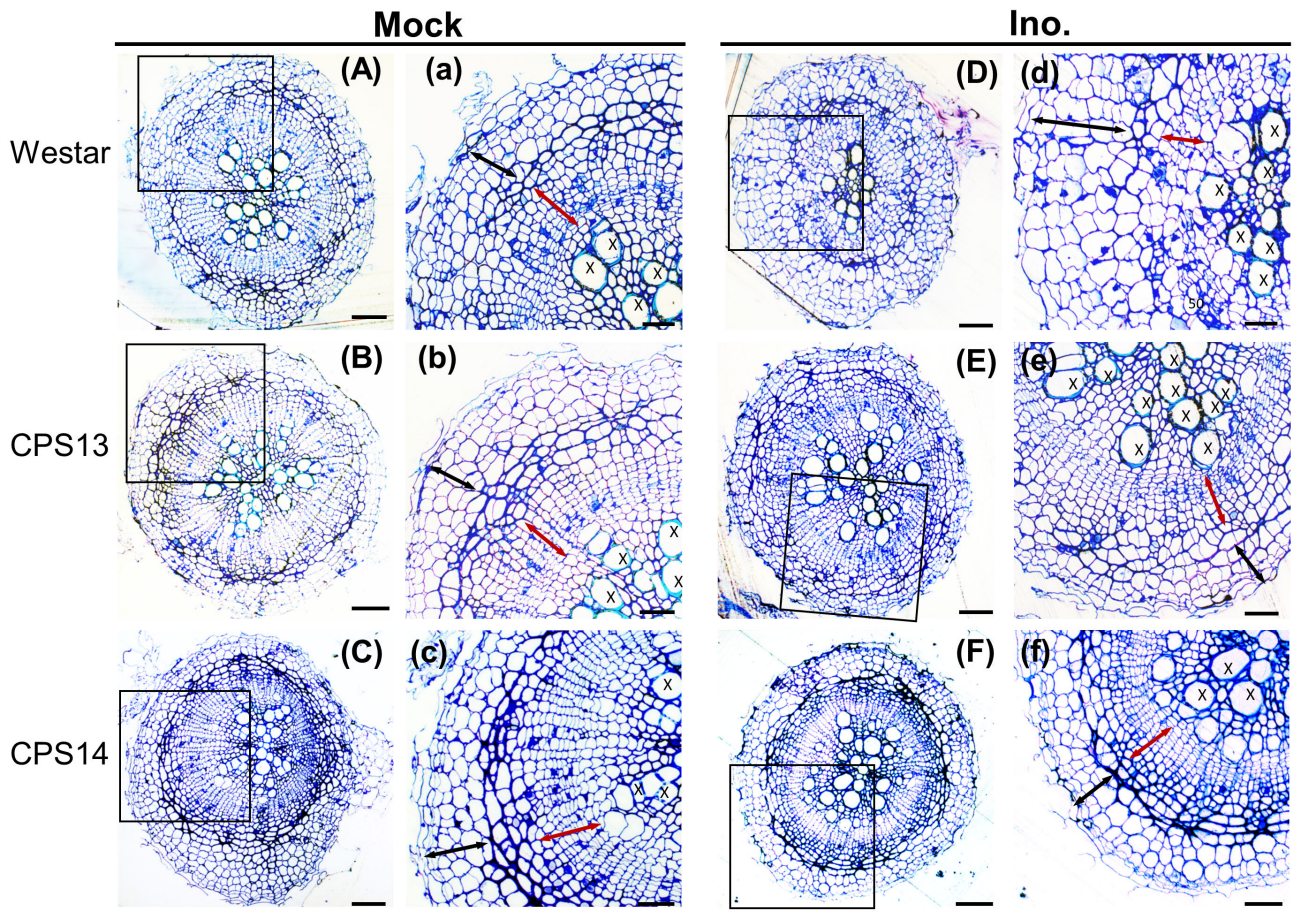
Cortical and xylem anatomical changes in susceptible (Westar) and resistant (CPS13 and CPS14) during secondary infection at 12 dpi. Cross sections of canola roots were stained with toluidine blue to show anatomical changes of roots in response to *P. brassicae* infection **(A–F)**, with insets (a–f) showing higher magnification of regions of cortical cells (black arrows), endodermis (the darker blue layer between black and red arrows) and vascular cambium (red arrows) from the squares on the root cross sections. Label X, xylem. Scale bars represent 100 mm for root cross sections and 60 mm for the insets.

**Figure 3:**
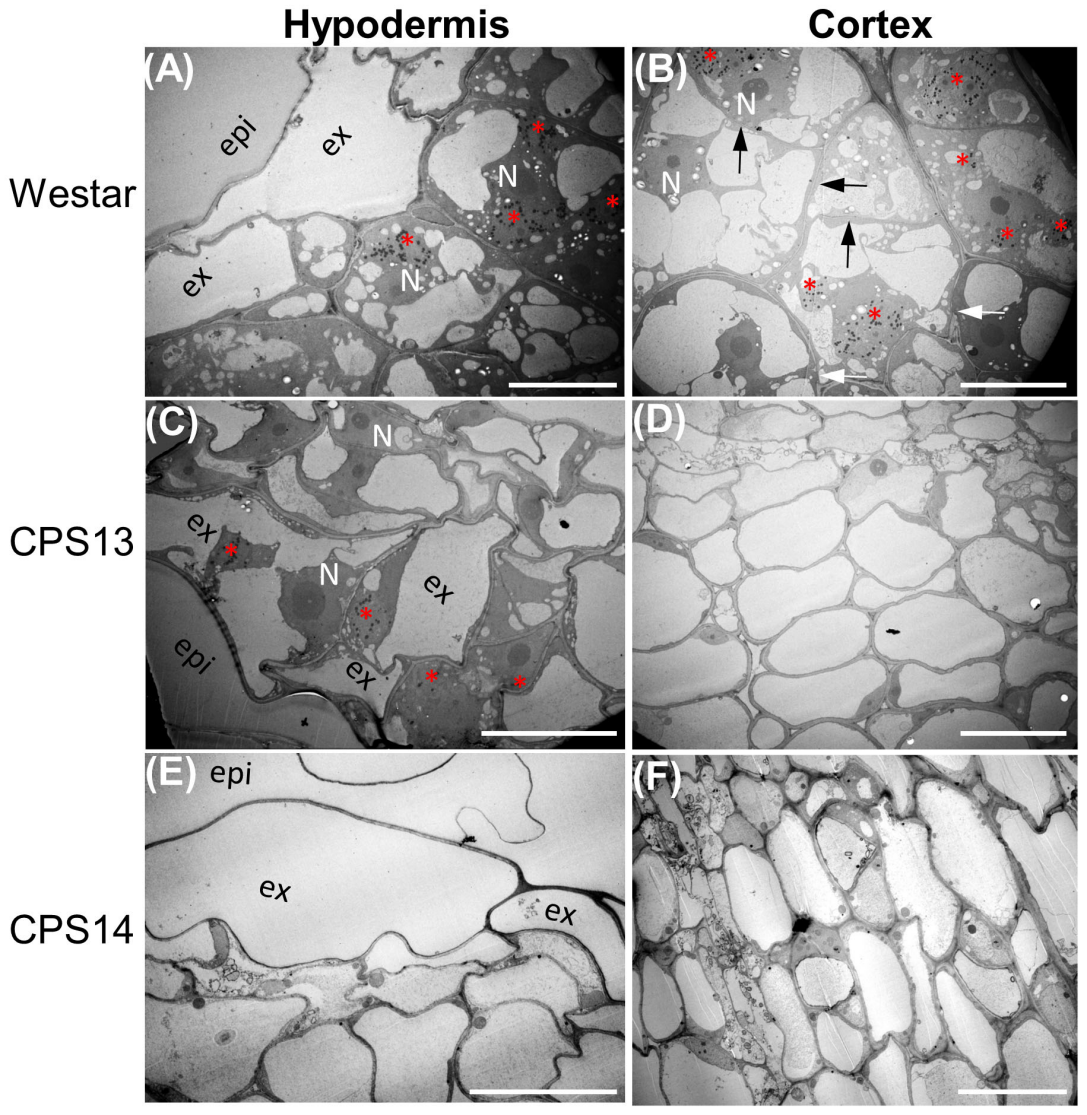
Colonization of hypodermis and cortex of susceptible **(A, B)** resistant **(C–F)** canola roots by *P. brassicae* at 12 dpi. Pathogen structures are captured by electron-dense lipid droplets in transmission electron micrographs as black dots and multinuclei in a host cell (red asterisks). **(A)** Multi-nucleated plasmodia in exodermal cells of Westar, with enlarged host nuclei at a central position and accumulation of host starch granules. **(B)** Multi-nucleated plasmodia in cortical cells of Westar with thinned (black arrows) and raptured (white arrows) cell walls. **(C)** Multi-nucleated plasmodia in exodermal cells of CPS13 roots; note that host starch granules were not present, and enlarged host nuclei were observed in both infected and uninfected cells. **(D)** Noninfected cortical cells in inoculated CPS13 roots. **(E, F)** Plasmodia is not found in either hypodermis or cortex of CPS14. Labels epi, epidermis; ex, exodermis; N, host nucleus. Scale bars represent 20 µm.

Secondary or cortical infection generally peaks at about 14 dpi (Sharma et al., 2011; Chu et al., 2014), and is considered the critical stage for host defense responses (Zhang et al., 2023). At 12 dpi, the cortical tissue of Westar was successfully penetrated by *P*. *brassicae* (**Figures 2D**, **3B**), shrinking the xylem tissue compared with uninoculated Westar **Figure 3A**). In contrast, the root anatomy of resistant varieties remained unchanged in response to infection (**Figures 2B, C, E, F**). Transmission electron microscopy (TEM) showed that the penetration of cortical cells by *P. brassicae* was halted in hypodermis of resistant varieties (**Figure 3**). Enlarged nuclei were frequently observed in infected cells of both susceptible and resistant varieties (**Figure 3**). However, the accumulation of host starch granules was only observed in infected cells of the susceptible variety. Additionally, CWs of infected cortical cells often ruptured in the susceptible variety, potentially facilitating plasmodium infection and colonization of neighboring cells (**Figure 3B**).

**Figure 4:**
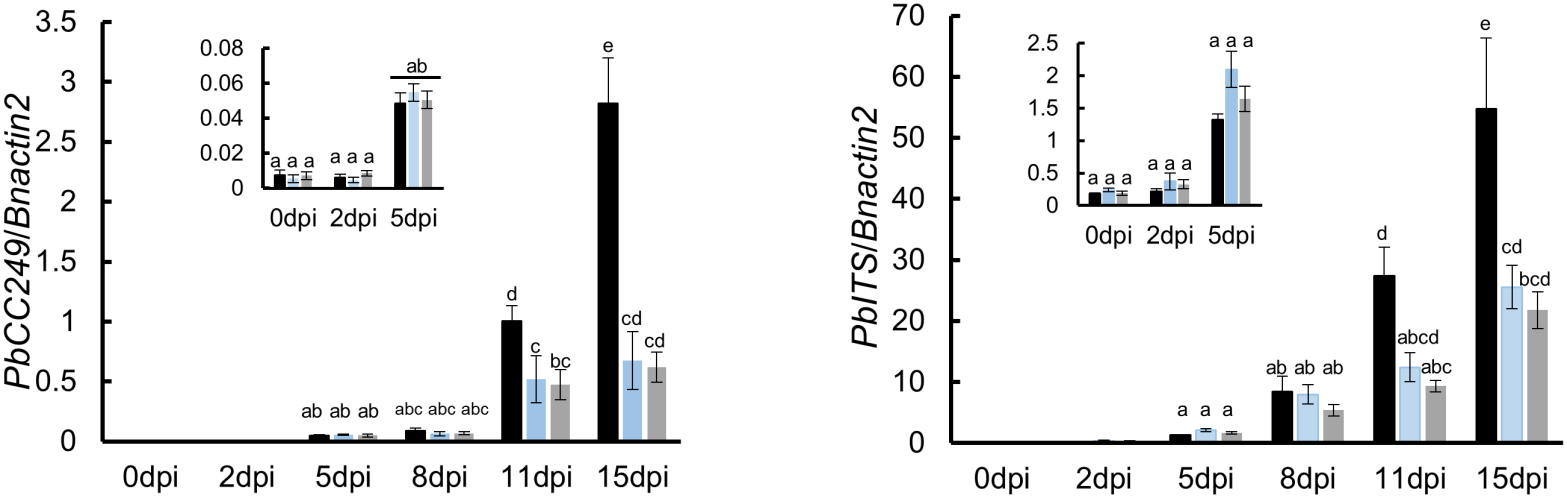
Quantification of *P. brassicae* biomass in roots of susceptible and resistant varieties with qRT-PCR over the time course of infection between 0 and 15 dpi. The amount was based on the relative quantity of mRNA from the *P. brassicae* ITS (*PbITS*) gene (left panel) and the *P. brassicae* ribosomal protein (*PbCC249*, right panel) gene, compared respectively to the *B. napus* actin (*Bnactin*) gene. Black, blue and grey bars ± SD (N = 3) represent Westar, CPS13 and CPS14 in these tests. Means with the same letter(s) within the same sampling date did not differ (*P* > 0.05, HSD test).

To complement the observations during primary and secondary infection processes, the *P. brassicae* biomass in root tissues was quantified using qRT-PCR in a time course until subtle swellings became clearly visible on Westar (15 dpi). No difference was found between susceptible and resistant varieties during the primary (root hair) infection, while substantially less amounts of *P. brassicae* were found in resistant CPS13 and CPS14 during secondary infection between 11 and 15 dpi compared with susceptible Westar (**Figure 4**). Additionally, the expression of *PR1, PR2* and *PR3* genes was also measured in the time course with the qRT-PCR, and infected roots showed similarly increased expression of these marker genes, relative to mock controls, in both susceptible and resistant varieties between 11 and 15 dpi (**Supplementary Figure S2**). In mock-inoculated roots, however, the expression of these PR genes was higher in resistant varieties than in Westar.

### Change in CW components during secondary infection

Fourier transform mid-infrared (FT-MIR) microspectroscopy was utilized to analyze plant CWs *in muro*, eliminating complicated sample preparation. The measurements were also tissues specific to minimize heterogeneity of CWs associated with different cell types (Somssich et al., 2016). Separate analyses were applied to CW components of cortex and endodermis, respectively, where *P. brassicae* infection often appeared to be halted in resistant varieties. Statistical analysis was carried out for absorbances in the fingerprint region (1800–900 cm^-1^) related to primary CW compounds. Using PCA of spectral features within the fingerprint region, we found a difference between infected and mock-control plants. The first component (PCA1) accounted for more than 79% of the variance in the original spectroscopic data. The analysis also showed a clear separation between the CW features of infected cortical and endodermal cells in Westar roots compared to mock controls (**Supplementary Figure S3**), indicating significant chemical changes induced by infection. Furthermore, a distinction was observed between susceptible and resistant canola following infection in the cortical layer (**Supplementary Figure S3**). As a result, we aimed to explore the dynamics of CW components in response to *P*. *brassicae* infection in the following section.

### The localization and content of CW components - FT-MIR analysis

**Figure 5:**
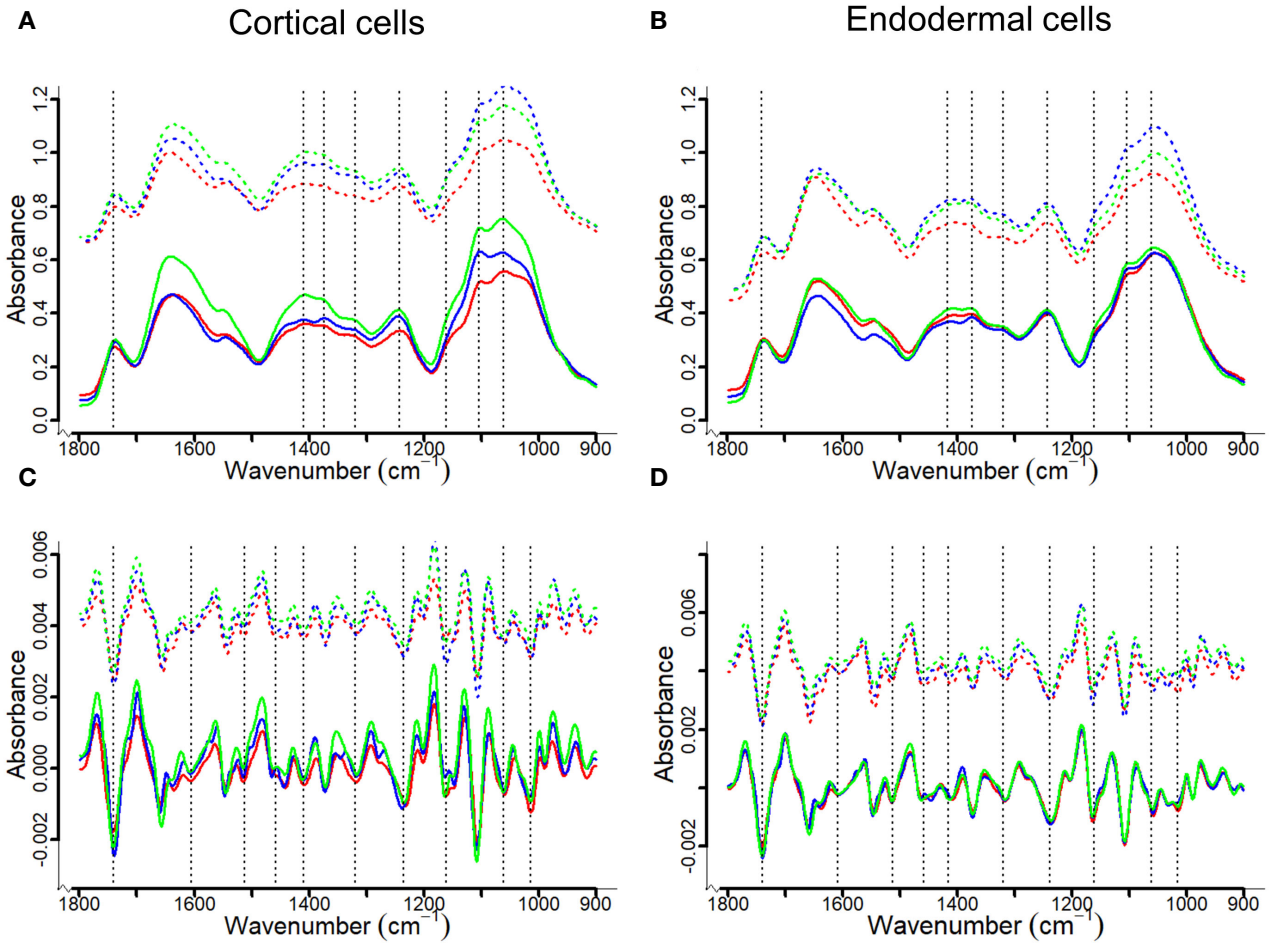
Averaged FT-MIR (upper panel) and their corresponding second derivation (lower penal) absorbance spectra for cortical and endodermal cell walls of canola roots. The spectra were offset to demonstrate differences between control (solid lines) and inoculated (dash lines) groups. The red, blue and green lines represent Westar, CPS13 and CPS14, respectively. Based on literature (Largo-Gosens et al., 2014; Kumar et al., 2016; Lahlali et al., 2017; Türker-Kaya, 2017; Bock and Gierlinger, 2019; Brar et al., 2019; Beć et al., 2020), three band areas (1615–1595 cm^-1^, 1520–1505 cm^-1^ and 1340–1300 cm^-1^) can be assigned to lignin, each potentially corresponding to S-, G- or H-type lignin (inconclusive). As only a small peak could be observed for the area 1340–1300 cm^-1^ **(A, B)**, second derivatives of the spectra were calculated to improve the visualization of 1615–1595 cm^-1^ and 1520–1505 cm^-1^ band areas **(C, D)**.

FT-MIR spectral profiles of the fingerprint region in cortical and endodermal cells showed that the most characteristic features visible are related to the functional chemical groups associated with the plant CW (**Figure 5**, **Table 1**), including cellulose, hemicellulose and pectin. The peak at 1061 cm^-1^ corresponded to C-O-C vibrations or cellulose (Largo-Gosens et al., 2014; Lahlali et al., 2017; Aubry et al., 2022), while the peak at 1246 cm^-1^ could be attributed to C=O and C-O vibration in pectin and hemicellulose (Le Hir et al., 2015; Lahlali et al., 2017; Türker-Kaya, 2017; Dinant et al., 2019; Beć et al., 2020). Carbonyl compounds with C=O groups would typically correspond to ester linkages; the presence of esterified pectin or C=O stretching for acetyl groups was clearly observed in the region at 1740 cm^-1^ (Le Hir et al., 2015; Lahlali et al., 2017; Türker-Kaya, 2017; Brar et al., 2019; Beć et al., 2020). The C=C stretching of the aromatic ring at the peaks 1510 cm^-1^ and 1595 cm^-1^ is a known marker for lignin (Lupoi et al., 2015; Lahlali et al., 2017; Türker-Kaya, 2017; Dinant et al., 2019; Beć et al., 2020). Additionally, absorbances at the peak 1330 cm^-1^ would correspond to S-mode lignin (Largo-Gosens et al., 2014; Lupoi et al., 2015).

Pronounced differences were observed in FT-MIR spectra between resistant and susceptible varieties following infection; there was a decrease in band intensity, reflected by decreased peak height in inoculated Westar in the fingerprint region, suggesting a decay in CW (**Figures 5A, B**). Second derivatives of the spectra were calculated to improve the visualization of closely spaced vibrational bands, enabling the identification of two functional groups associated with the aromatic ring of lignin: One centered around 1608 cm^-1^ within an integrated area of 1615–1595 cm^-1^, and another around 1512 cm^-1^ within an integrated area of 1520–1505 cm^-1^ (**Figures 5C, D**).

**Figure 6:**
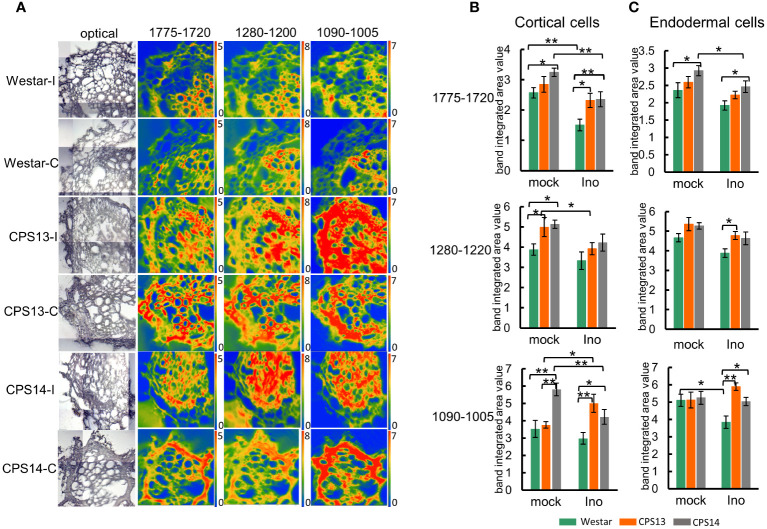
Changes in pectin, hemicellulose and cellulose following *P. brassicae* infection measured with FT-MIR microspectroscopy at 12 days post inoculation (dpi). **(A)** The chemical maps display the concentration of pectin, hemicellulose and cellulose in root cross-sections. The false-color images represent their content in cell walls; the hotter the color, the greater the concentration. Note that the same scale of absorbance was used for comparison. The intensity scales for band areas 1775–1720 cm^-1^, 1280–1200 cm^-1^ and 1090–1005 cm^-1^ are 0–5, 0–8 and 0–7, respectively. **(B, C)** Numerical variations of the band area assigned to pectin, hemicellulose and cellulose for mock and inoculated groups in cortical **(B)** and endodermal cells **(C)**. Two-way ANOVA with multiple comparison *post-hoc* test was used for analysis of data. Values represent mean ± SD. “* and **” indicate differences between varieties are significant at *P <*0.05 and 0.01 (Contrast, “emmeans” package), respectively. Labels I/Ino: inoculated, C/mock: mock control.

**Figure 7:**
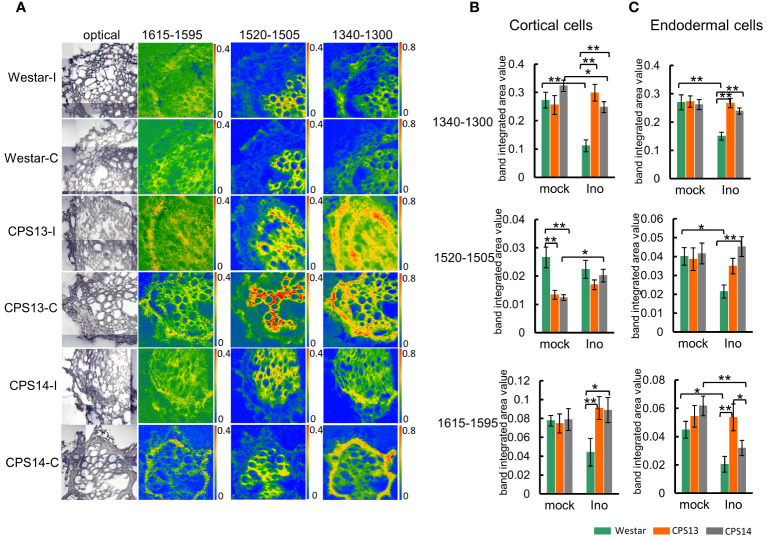
Changes in lignin compositions responding to *P. brassicae* infection measured with FTIR microspectroscopy at 12 dpi. **(A)** The chemical maps display the concentration of the lignin component in root cross-sections. The false-color images represent lignin in the cell walls and the hotter the color, the greater the concentration. Note the same scale of absorbance used for comparison (the intensity scales for band areas 1615–1596 cm^-1^, 1520–1505 cm^-1,^1340–1300 cm^-1^ are 0–0.4, 0–0.4 and 0–0.8 respectively). **(B, C)** Numerical variations of the band area assigned to lignin for the mock and inoculated groups in cortical **(B)** and endodermal cells **(C)**. Two-way ANOVA with multiple comparison *post-hoc* test was used, and the values represent mean ± SD. “* and **” indicate differences between varieties are significant at *P*<0.05 and 0.01 (Contrast, “emmeans” package), respectively. Labels I/Ino: Inoculated, and C/mock: mock control.

False color images were created to localize the distribution of key CW components, showing different patterns for cellulose, hemicellulose, pectin, and lignin (**Figures 6**, **7**). Integrated specific band areas under particular regions, considered as indicators of CW components, were also calculated and numerical variations analyzed. The contents of cellulose, hemicellulose, and pectin observed in non-inoculated resistant varieties, particularly in CPS14, were higher than those in Westar, as reflected by the band intensity (**Figure 5A**) and integrated band areas (**Figure 6**) in cortical cells. In contrast, a decrease in these CW components was observed in cortical layers of *P*. *brassicae*-inoculated roots, as indicated by chemical mapping and data statistics in both resistant and susceptible varieties (**Figures 6A, B**). However, no significant changes were found with these compounds in endodermis regardless of inoculation (**Figures 5B**, **6C**), suggesting CW modifications occurred mainly at the pathogen contact sites on cortex.

The chemical absorbance map obtained from the band area 1520–1505 cm_1_ showed a precise xylem-related location of the CW compounds, although it is outside the area of interest (**Figure 7A**). Statistical analysis also indicated that the amount of lignin associated with the peak area 1520–1505 cm_1_ was much lower in cortical and endodermal cells (**Figures 7B, C** top panel). Similar absorbance maps for the peak areas 1340–1300 cm^-1^ and 1615–1595 cm^-1^ were also acquired, showing a higher concentration with cortical and endodermal cells of resistant varieties relative to Westar (**Figures 7B, C**) after infection, suggesting CW lignification plays a role in clubroot resistance. Following *P. brassicae* infection, lignin content decreased in cortical and endodermal cells of Westar, but increased or remained steady in resistant varieties.

### Lignin deposition in exodermal cells

**Figure 8:**
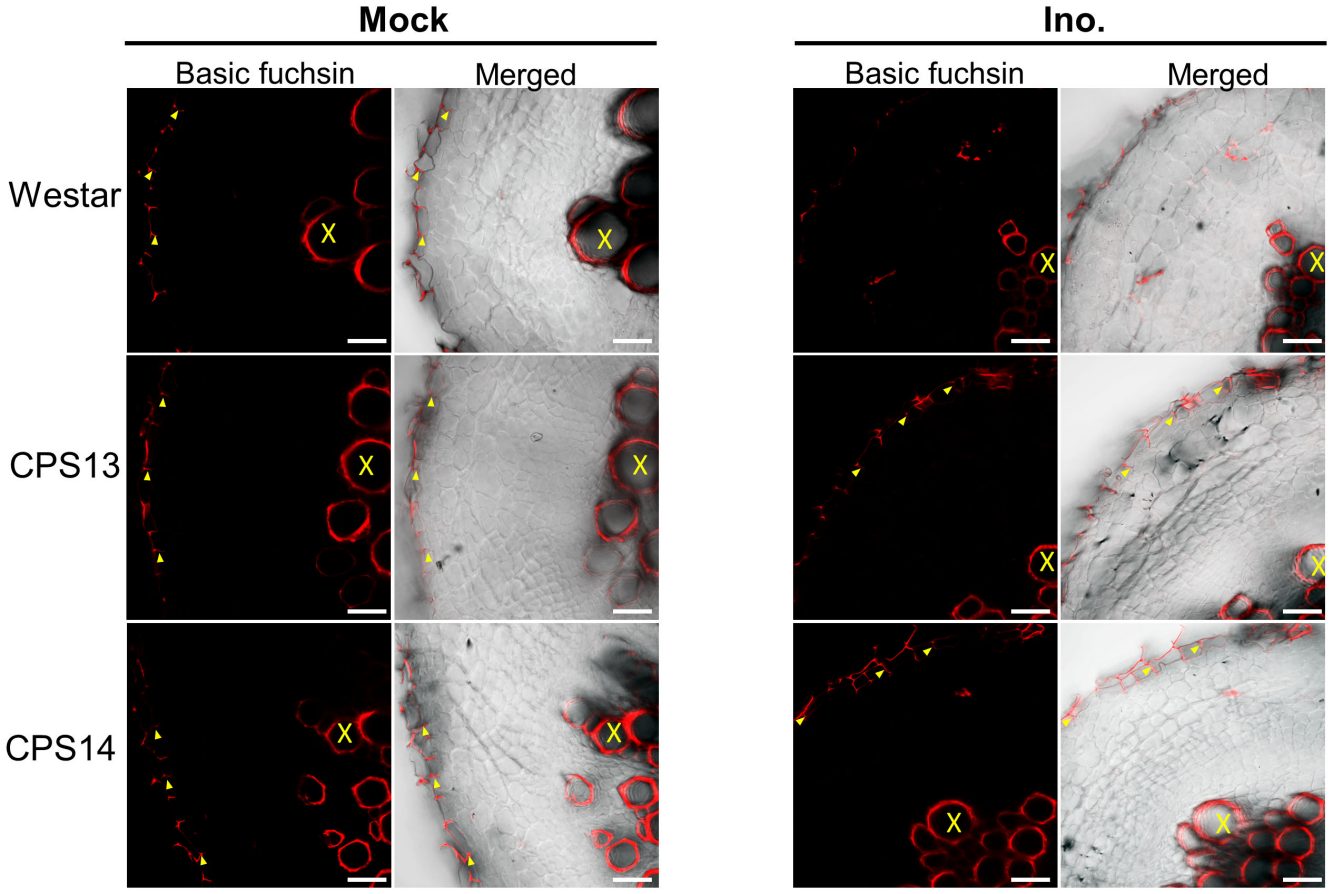
Lignin deposition in exodermal cells in cross section of susceptible (Westar) and resistant (CPS13, CPS14) canola varieties in response to *P. brassicae* infection stained with basic fuchsin at 12 dpi. The red color shows the lignin signal in CW. No lignin signal was detected on the anticlinal cell walls of inoculated Westar roots. Labels X: Xylem vessels, yellow arrowhead: Lignification in anticlinal cell walls of exodermis. Scale bars represent 50 µm.

During root infection, *P. brassicae* must pass through epidermal and exodermal layers before reaching the cortical tissue. However, Casparian strips and suberin deposition in the exodermis can act as a physical barrier to infection (Rich et al., 2014). At 12 dpi, lignin staining revealed band-like streaks of lignin on the anticlinal side of the exodermis (**Figure 8**, left panel), resembling the location where the Casparian band would typically be present in non-inoculated samples (Meyer and Peterson, 2013). Additionally, solid lignin signals were also observed in CWs of xylem tissue. During the infection, however, the lignification was generally not seen in exodermal cells of Westar (**Figure 8**, right top panel). By contrast, local lignin accumulation was consistently observed in the outer CWs of exodermal cells (**Figure 8**, right bottom panel), which generally coincided with aborted cortical infection in resistant varieties.

### Expression of genes involved in general lignin biosynthesis induced by infection

**Figure 9:**
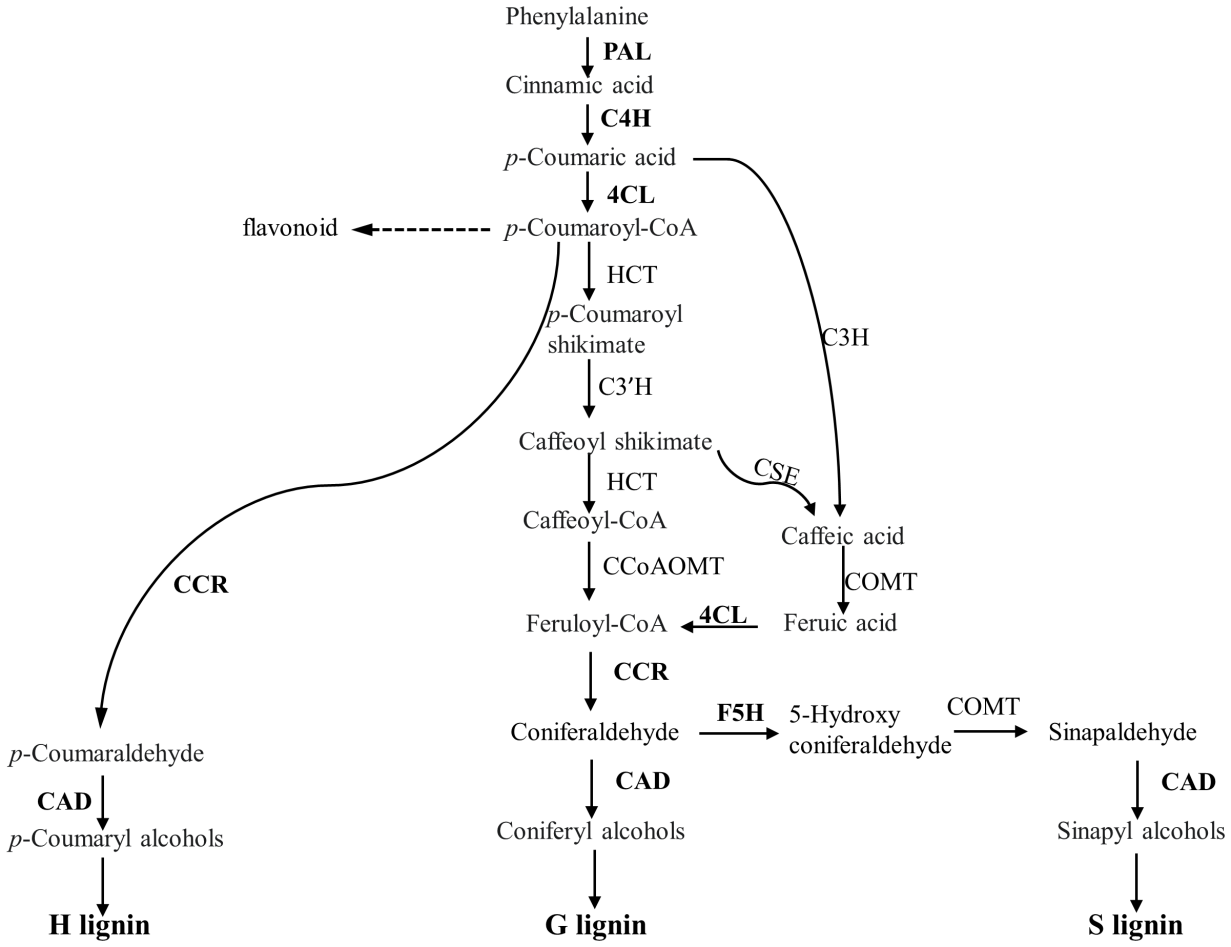
Overview of phenylpropanoid pathway leading to monolignol biosynthesis. The expression of the genes encoding the enzymes in bold was examined using qRT-PCR following inoculation. PAL, Phenylalanine ammonia lyase; C4H, Cinnamate 4-hydroxylase; 4CL, 4-coumaroyl-CoA ligase; HCT, p-hydroxycinnamoyl-CoA:shikimate p-hydroxycinnamoyl transferase; CCoAOMT, Caffeoyl-CoA *O*-methyltransferase; CSE, Caffeoyl shikimate esterase; CCR, Cinnamoyl-CoA reductase; F5H, ferulate 5-hydroxylase.

**Figure 10:**
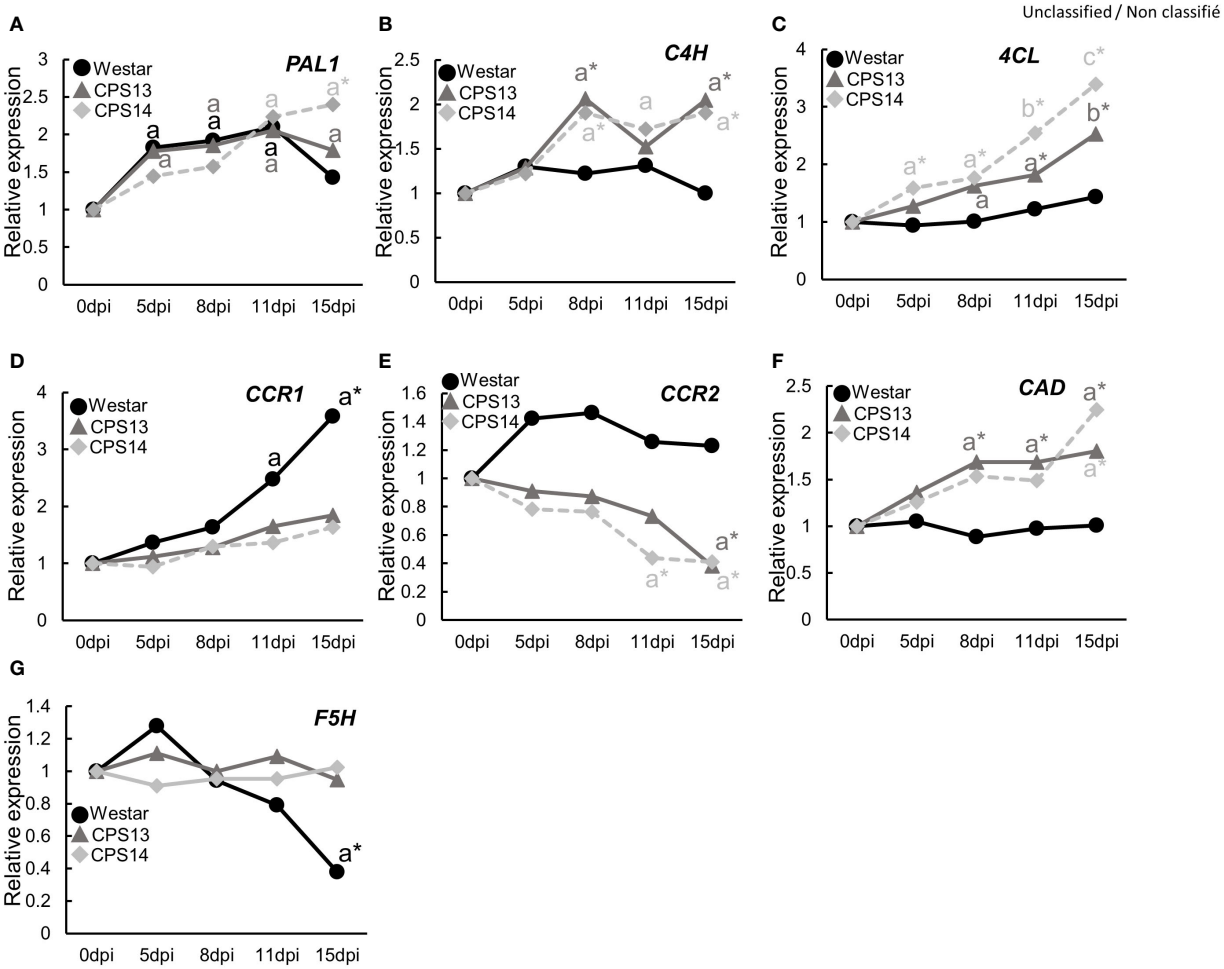
Expression of genes in the general phenylpropanoid pathway, including PAL1 **(A)**, C4H **(B)**, 4CL **(C)**, CCR1 **(D)**, CCR2 **(E)**, CAD **(F)** and F5H **(G)**, in the *P. brassicae* infected roots of susceptible (Westar) and resistant (CPS13, CPS14) varieties at 0, 5, 8, 11 and 15 days post inoculation (dpi). Three biological replicates, each consisting of root samples from five plants, were used for each variety, and three technical replicates were used for the analysis of each biological replicate. All data were normalized to the expression of *ACTIN2*, and the fold induction value of all genes was calculated relative to the expression level of inoculated plants at 0 dpi. The means with the letter “a” indicates a significant difference (*P <*0.05) relative to the value at 0 dpi, while asterisks indicate a significant difference (*P* < 0.05) of resistant varieties compared to Westar for those sampling dates. Details on statistical analysis are included in **Supplementary Table 2** generated by the Compact Letter Display function in the Emmeans package of RSdusio.

As the above experiments showed potential role of CW lignification in the resistance, we reasoned that the expression of genes involved in phenylpropanoid pathway could be upregulated, including those encoding major enzymes in lignin biosynthesis pathways (**Figure 9**), such as phenylalanine ammonia lyase1 (*PAL1*), cinnamate 4-hydroxylase (*C4H*), 4-coumarate: CoA ligase3 (*4CL*), and cinnamyl alcohol dehydrogenase (*CAD*). They were measured with qRT-PCR (**Figure 10**; **Supplementary Table 2**). The expression of *PAL1* might be slightly induced overtime in both resistant and susceptible varieties (**Figure 10A**). The expression of *C4H*, *4CL* and *CAD* increased specifically in both resistant varieties from 8 dpi, relative to susceptible Westar, reaching the expression peak at 15 dpi (**Figures 10B, C, F**). However, the expression of *CCR1* was induced more substantially in Westar over time (**Figure 10D**), while the expression of *CCR2* appeared to be suppressed in resistant varieties compared to the base value at 0 dpi (**Figure 10E**). The expression of *F5H* (Ferulate/coniferaldehyde 5-hydroxylase) showed an opposite trend of *CCR2*, with a decrease in Westar but stable expression levels in both resistant varieties over time (**Figure 10G**).

## Discussion

For years, we have been working to decipher the molecular mechanisms underlying clubroot resistance in canola. Our studies have included the roles of jasmonic acid (JA) signaling and callose deposition (Chu et al., 2014), the activation of mitogen-activated protein kinases driven by a ubiquitin-linked proteasome promoted by JA (Song et al., 2016), the upregulation of critical genes in the phenylpropanoid pathway (Lahlali et al., 2017), and the enhanced functionalities of both PTI and ETI associated with *Rcr1* and *Crr1^rutb^* (Wen et al., 2024). The current study utilized histological, microscopic and microspectroscopic analyses following *P*. *brassicae* infection to complement prior studies by focusing on the cellular and structural changes mediated by a single (*Rcr1*) or stacked (*Rcr1* + *Crr1^rutb^*) CR genes. We observed that resistant varieties, carrying either single or stacked CR genes, had similar defense responses against *P*. *brassicae* infection, with thickened and more lignified cortical cell walls than susceptible controls, which may contribute to halting pathogen ingress into cortical tissues. The observed cell wall modifications may be related to the activation of a range of PTI responses for these CR genes (Wen et al., 2024). Both *Rcr1* and *Crr1^rutb^* also encode TIR-NBS-LRR proteins, a critical ETI factor for clubroot resistance (Ueno et al., 2012; Hatakeyama et al., 2013; Kopec et al., 2021; Hu et al., 2024). Despite this, a hypersensitive reaction was not observed throughout the current study. This suggests that the activation of genes involved in JA/phenylpropanoid pathways and PTI/ETI (Chu et al., 2014; Song et al., 2016; Lahlali et al., 2017; Wen et al., 2024) trigger a cascade of structural and possibly metabolic changes, restricting pathogen ingress into the cortical tissues of resistant canola. During the infection process, clubroot resistance may even involve a temporal coactivation of both JA and SA signaling pathways (Lemarié et al., 2015), rendering sophisticated modes of action against the pathogen.

In Arabidopsis, activation of SA signaling increased the expression of *PR2* and *PR5* genes in a partially resistant accession (Lemarié et al., 2015), while in *B. napus*, *PR1*, *PR2* and *PR4*, were consistently up-regulated in both host genotypes during secondary infection by *P. brassicae* (Galindo-Gonzalez et al., 2020). It was a bit surprising that none of the *PR* marker genes measured was differentially activated in roots of inoculated resistant varieties relative to the susceptible control (**Supplementary Figure S2**). In our transcriptome and proteome studies earlier, only JA signaling, not SA, was activated by the CR gene *Rcr1* (Chu et al., 2014), which initiates a mitogen-related protein kinases stimulated by JA (Song et al., 2016), likely leading the upregulation of genes involved in the phenylpropanoid pathway (Lahlali et al., 2017). These suggest PR proteins are not involved in clubroot resistance conferred by *Rcr1* or *Crr1^rutb^*. The accumulation of starch granules observed in the exodermal cells of infected Westar (**Figure 3**) could provide additional carbon nutrients, supporting the pathogen during colonization and proliferation in roots of susceptible hosts.

### Cortex infection is halted at exodermis in resistant canola

Our understanding of CR process mediated by resistance loci has mostly been based on the information from model plant Arabidopsis (Ochoa et al., 2023; Wang et al., 2023), including reduction of resting-spore germination and root-hair infection (Rashid et al., 2013), and prevention of plasmodium establishment or maturation in cortical cells (Kobelt et al., 2000; Tanaka et al., 2006; Deora et al., 2012, Deora et al, 2013; Liu et al., 2022). In this study, we aimed to establish a timeframe for resistant interactions in canola using microscopic, molecular and spectroscopic techniques. Our findings showed that the effect was more pronounced during cortical infection. Although these results are similar to those reported previously (Deora et al., 2013; Summanwar et al., 2019; Liu et al., 2020, Liu et al, 2022; Ochoa et al., 2023; Wang et al., 2023), they also help pinpoint the timing and location of the inhibition during secondary infection in resistant canola. By comparing the root anatomy and ultrastructure, our findings show that *P*. *brassicae* can still penetrate the hypodermis of resistant canola. However, further spread into cortical cells is effectively blocked by the exodermis, as demonstrated by the results at 12 dpi (**Figure 3**). Exodermis is an outer cortex layer, which is often reinforced by thickened CWs with deposition of a Casparian strip and suberin lamella like endodermis (Meyer and Peterson, 2013; Cantó-Pastor et al., 2024), which appears to trap the pathogen in resistant canola. These results are also backed up by much less amounts of *P. brassicae* biomass found in roots of resistant CPS13 and CPS14 compared to Westar, during secondary infection, due to much reduced colonization/proliferation of *P*. *brassicae* in the cortex.

### Characterizing root CWs in response to *P. brassicae* infection

In this study, we observed thicker outer hypodermis CWs in resistant varieties and potential degradation of cortical CWs in Westar during secondary infection (**Figure 3**). As this pathogen does not appear to possess genes involved in CW degradation or modification (Schwelm et al., 2015; Rolfe et al., 2016), it is likely that the host orchestrates the differential CW characteristics in response to *P. brassicae* entry and spread. Other studies have also shown that secondary plasmodia could spread from infected cortical cells of susceptible plants to neighboring cells via ‘broken’ CWs (Donald et al., 2008; Deora et al., 2013). Quantifying CW constituents, especially in conjunction with transcriptome analysis, may provide insights into key elements involved in forming physical barriers against infection. In this study, both measurements indicated a role for lignin. Comprehensive transcriptome studies have also shown differential CW responses between susceptible and resistant plants during secondary infection of clubroot, particularly around 14 dpi (Chu et al., 2014; Zhang et al., 2016; Irani et al., 2018; Badstöber et al., 2020; Galindo-Gonzalez et al., 2020), which generally coincide with the resistance against the cortical infection.

The clubroot pathogen traverses the root of susceptible plants radically during the infection process, beginning with the outermost epidermis and followed by the invasion of exodermis, cortex and endodermis (Liu et al., 2022; Javed et al., 2023). Individual tissue layers may perceive and respond to the invasion in an unique and distinct manner (Fröschel et al., 2021; Kawa and Brady, 2022). Our earlier study using FT-MIR (Lahlali et al., 2017) showed that CW modifications might have occurred in both susceptible and resistant varieties in response to *P. brassicae* infection based on the results of bulked root samples. Cell-type specific mapping and quantification of CW components in the current study revealed intrinsic site-specific CW modification induced by the pathogen, which may act as physical barriers in resistant canola. FT-MIR microspectroscopy demonstrated that polysaccharide contents in CWs, especially the amount of cellulose (integrated area 1090–1050 cm^-1^), hemicellulose (1280–1200 cm^-1^) and pectin (1775–1720 cm^-1^) in cortical cells, could be clearly differentiated between the susceptible and resistant varieties (**Figure 6B**). Resistant varieties contained more polysaccharide cell wall contents that were reduced by pathogen infection in both susceptible and resistant reactions. A recent study with Raman spectroscopy also showed a decrease in cellulose, hemicellulose and pectin during gall development in susceptible plants (Stefanowicz et al., 2021). In our previous study, genes encoding pectin lyase, which plays a significant role in pectin degradation, and genes encoding expansin, responsible for disrupting the function of cellulose by loosening its microfibrils, were downregulated in resistant *B. rapa* carrying the CR gene *Rcr1* (Chu et al., 2014). However, some studies have found that the expression of genes involved in hemicellulose and pectin metabolism were downregulated during the infection of both susceptible and resistant plants by *P*. *brassicae* (Zhang et al., 2016; Galindo-Gonzalez et al., 2020). Therefore, the role of these polysaccharides in clubroot resistance may vary depending on the host.

While polysaccharides often changed little in the CWs between resistant and susceptible plants, especially in endodermal cells (**Figures 7B, C**), there was a strong indication that CW lignin is degraded in both cortical and endodermal cells of susceptible plants following infection. This was particularly evident from the band areas 1615–1590 cm_1_ and 1340–1300 cm_1_ (**Figures 7B, C**).These results are slightly different from those of our earlier study (Lahlali et al., 2017) where lignin content increased in roots of both resistant and susceptible plants inoculated with the clubroot pathogen. The discrepancy is likely due to the FT-MIR measurement on bulked root tissues used previously that could be affected by the heterogeneity CW contents in different cells (Somssich et al., 2016). The cryo-sectioned root samples used in this study can analyze CW components for specific root tissues, mitigating potential dilution or masking of differential lignification signals from bulk samples. These cell specific FT-MIR CW polysaccharide and lignin markers, as well as FPA imaging, can be used for characterizing or differentiating CR genotypes in research.

### Lignin composition and content play a role in clubroot resistance

FT-MIR results suggested that CW lignification plays a role in clubroot resistance, which prompted us to look further into some details. Lignin forms a mechanical barrier, which may trap pathogens at the infection site (Lee et al., 2019), decreasing nutrient access by pathogens (Miedes et al., 2014). With basic fuchsin staining, we showed that lignin in exodermal cells might somewhat be degraded by *P. brassicae* during the early stage of secondary infection in the susceptible Westar, where the expression of genes involved in lignin biosynthesis was not activated by the infection either (**Figures 9**, **10**). Nonlignified passage cells in exodermis have been shown to serve as gateways for arbuscular mycorrhizal fungi to reach cortical tissues of roots (Sharda and Koide, 2008; Rich et al., 2014). In contrast, several genes involved in the general phenylpropanoid pathway (*C4H*, *4CL* and *CAD*) were up-regulated exclusively in the resistant varieties (**Figure 10**), which also corresponded to the transcriptomic data from an earlier study by Chu et al. (2014) that showed genes involved in lignin biosynthesis and transcriptional factors regulating the expression of lignin synthesis genes were induced by the CR gene *Rcr1*. The induction of these genes was corroborated by FT-MIR results that showed increased lignin content in resistant varieties relative to Westar under inoculation conditions. These findings are generally consistent with the measurements of pathogen biomass, PR gene expression, and CW compounds, which collectively showed that lignin and its associated phenolics accumulated in resistant cultivars at the onset of cortical infection.

Interestingly, genes encoding enzymes in downstream of the phenylpropanoid pathway and catalyzing the biosynthesis of specific monolignols showed distinct expression patterns over the time course (**Figure 10**); increased expression of *CCR1* and *F5H* in Westar may suggest a reduced S (guaiacyl)/G (syringyl) lignin ratio, and a decreased expression of *CCR2* in resistant varieties may suggest the accumulation of G- and S-lignin at the expense of H (p-hydroxyphenyl) lignin. Working with clubroot-resistant *B*. *oleracea*, Zhang et al. (2016) found increased expression of genes associated with the phenylpropanoid biosynthesis pathway, some of which regulate the biosynthesis of G and S lignin. In a different study, higher S and H and lower G lignin amounts were found with common wheat resistant to Fusarium Head Blight when compared to susceptible durum wheat (Lionetti et al., 2015). In this study, absorbances at the peak 1330 cm^-1^ during the cortical/endodermal infection would correspond to S-mode lignin (Largo-Gosens et al., 2014; Lupoi et al., 2015) in the resistant varieties CPS13 and CPS14. It appeared that greater S/G lignin ratio has been more frequently related to disease resistance. However, results on the impact of lignin type to host resistance may vary depending on the disease; suppression of F5H also reduced the S/G ratio and increased the susceptibility to *Verticillium longisporum* (König et al., 2014). As the evidence is still scarce and not unequivocal, caution is required in generalizing the importance of S/G ratio; more data are needed. Taken together, our data suggest that elevated lignin accumulation play an important role in clubroot resistance; this increase may be activated particularly in exodermis via enhanced expression of genes involved in phenylpropanoid and branch pathways toward S-lignin units.

## Data availability statement

All relevant data is contained within the article: The original contributions presented in the study are included in the article/**Supplementary Material** and further inquiries can be directed to the corresponding author.

## Author contributions

JT: Conceptualization, Data curation, Investigation, Methodology, Software, Writing – original draft, Writing – review & editing, Validation. LQ: Writing – review & editing. CK: Conceptualization, Resources, Writing – review & editing. YW: Conceptualization, Writing – review & editing. GP: Conceptualization, Funding acquisition, Methodology, Project administration, Resources, Supervision, Writing – original draft, Writing – review & editing, Investigation.
