# Supplementary material for: Lignin accumulation in cell wall plays a role in clubroot resistance
Source: Front Plant Sci. 2024 Jul 23;15:1401265. doi: 10.3389/fpls.2024.1401265 (PMC11300216; doi:10.3389/fpls.2024.1401265)
Supplement: Supplementary file 3 [file DataSheet_1.pdf]

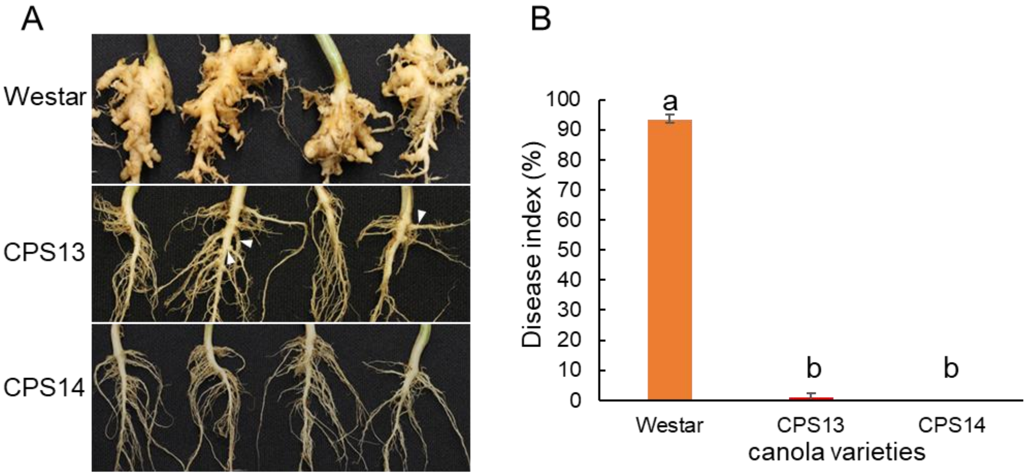

Supplementary Figure 1. Clubroot disease severity (DSI) on susceptible (Westar) and resistant (CPS13 and CPS14) canola at five weeks following the inoculation with *P. brassicae* pathotype 3H **(**5×10^6 spores/mL). (A) Representative symptoms; the arrowheads show small swellings on CPS 13. (B) mean DSI ± SD (N = 3) on the three canola varieties; different letters above the bars indicate statistical significance ($P < 0.05$, Tukey's HSD).

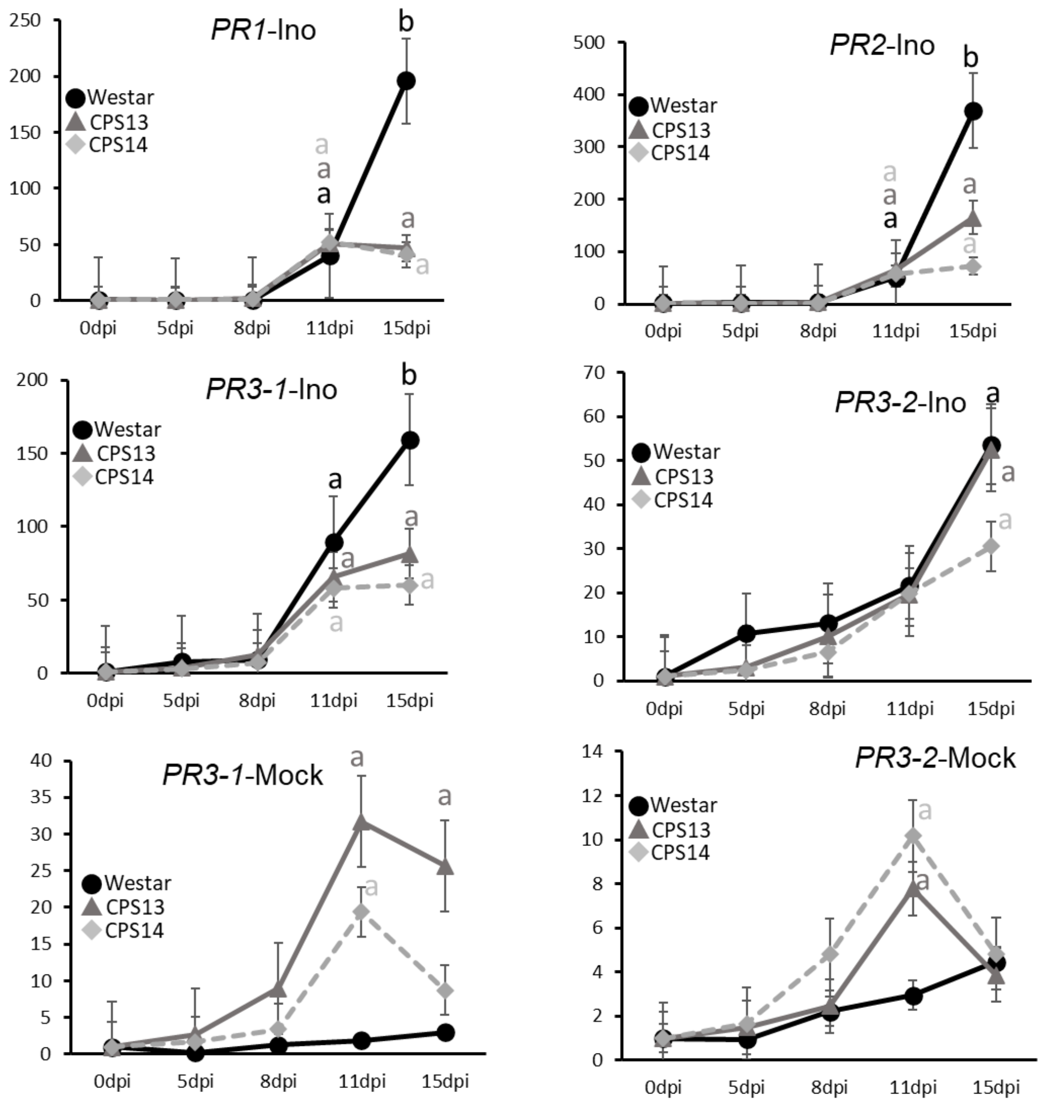

Supplementary Figure 2. Expression of pathogenesis-related (PR) genes following inoculation with *P. brassicae* in susceptible and resistant varieties for up to 15 dpi (N=3). All data were normalized to the expression of *ACTIN2* and fold induction value of calculated relative to the expression level of inoculated plants at 0 dpi (value set to 1). Values represent mean ± SD. Different letters indicate significant difference from 0 dpi ($P < 0.05$).

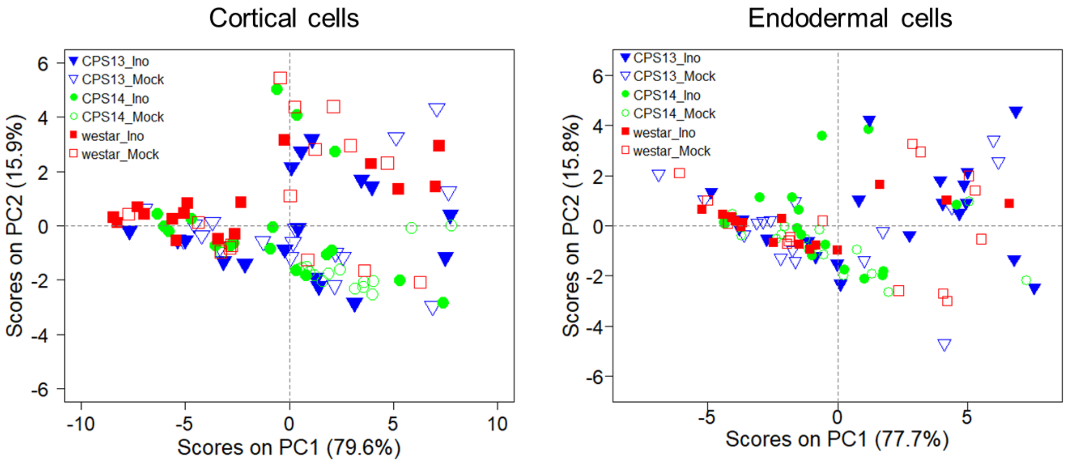

Supplementary Figure 3. Principal component analysis (PCA) plots of the transmission Fourier transform mid infrared (FT-MIR) spectra from cortical and endodermal cells of roots collected from three varieties, under mock (Mock) and inoculated (Ino.) conditions, within the spectral regions of 1800-900 cm^{-1} (fingerprint region). Score plot of the first principal components (PC1) versus the second principal components (PC2) are shown.
